# Cost-effectiveness analysis of AS04-adjuvanted human papillomavirus 16/18 vaccine compared with human papillomavirus 6/11/16/18 vaccine in the Philippines, with the new 2-dose schedule

**DOI:** 10.1080/21645515.2016.1269991

**Published:** 2017-01-11

**Authors:** Maria Julieta Germar, Carrie Purugganan, Ma. Socorro Bernardino, Benjamin Cuenca, Y-Chen Chen, Xiao Li, Georges Van Kriekinge, I-Heng Lee

**Affiliations:** a University of the Philippines College of Medicine, Philippine General Hospital, Metro, Manila, Philippines; b St Martin De Porres Charity Hospital, San Juan, Mandaluyong City, Philippines; c St Luke's Medical Center, Quezon City, Philippines; d Jose R Reyes Memorial Medical Center, Tondo, Manila, Philippines; e GSK, Singapore, Republic of Singapore; f GSK, Wavre, Belgium

**Keywords:** Two-dose, *Cervarix*™, cervical cancer, cost-effectiveness, *Gardasil*, human papillomavirus, Philippines, vaccination

## Abstract

Cervical cancer (CC) is the second leading cause of cancer death among Filipino women. Human papillomavirus (HPV) vaccination protects against CC. Two vaccines (AS04-HPV-16/18 and 4vHPV) are approved in the Philippines; they were originally developed for a 3-dose (3D) administration and have recently been approved in a 2-dose schedule (2D). This study aims to evaluate the cost-effectiveness of HPV vaccination of 13-year-old Filipino girls, in addition to current screening, in the new 2D schedule. An existing static lifetime, one-year cycle Markov cohort model was adapted to the Philippine settings to simulate the natural history of low-risk and oncogenic HPV infection, the effects of screening and vaccination of a 13-year-old girls cohort vaccinated with either the 2D-AS04-HPV-16/18 or 2D-4vHPV assuming a 100% vaccination coverage. Incremental cost, quality-adjusted life year (QALY) and cost-effectiveness were derived from these estimates. Input data were obtained from published sources and Delphi panel, using country-specific data where possible. Sensitivity analyses were performed to assess the robustness of the model. The model estimated that 2D-AS04-HPV-16/18 prevented 986 additional CC cases and 399 CC deaths (undiscounted), as well as 555 increased QALY (discounted), and save 228.1 million Philippine pesos (PHP) compared with the 2D-4vHPV. In conclusion, AS04-HPV-16/18 is shown to be dominant over 4vHPV in the Philippines, with greater estimated health benefits and lower costs.

## Introduction

Cervical cancer (CC), with an estimated yearly incident number of 6,670 cases and 2,832 deaths (year 2012),^1^ is the second most frequent cancer and the second leading cause of cancer death in the Philippines in women of all ages. Although CC can be managed successfully when detected at an early stage by regular screening, uptake of screening in the Philippines is low with a participation rate of around 8%.^2^ Approximately 75% of CC cases in the Philippines are diagnosed at a late stage and treatment is frequently unavailable, inaccessible or unaffordable.^2^ This contributes to the high mortality rate due to CC (age-standardised mortality rate: 7.5 per 100,000 women) in the Philippines.^1,2^

Persistent high-risk human papillomavirus (HPV) infection is a necessary cause of CC, with HPV detected in 99.7% of CC cases worldwide and certain risk factors in the presence of HPV infection may increase the risk of CC.^3,4^ More than 170 HPV types have been identified to date of which 40 HPV types infect the anogenital tract.^5,6^ Of these 40 types, 8 account for over 90% of CC cases worldwide.^7^ The most common oncogenic HPV subtypes are HPV-16 and HPV-18, which together account for approximately 70% of all CC cases worldwide.^7^ Non-oncogenic (low-risk) HPV types can cause low-grade lesions of the cervix and genital warts but are not causally linked to the development of cancer in humans.^6^ Vaccines against HPV exist and, by preventing HPV infections, vaccination may protect against CC. Typically, the vaccine is given to girls around the age of 12 or 13 y and ideally before the onset of sexual activity and thus before first exposure to cervical HPV infection. Two HPV vaccines are currently available and available to the public in many countries: (1) *Cervarix*™, an HPV-16/18 AS04-adjuvanted vaccine (AS04-HPV-16/18) protecting against HPV-16 and HPV-18 types; and (2) *Gardasil*, a HPV-6/11/16/18 L1 virus-like particle vaccine (4vHPV) protecting against 2 non-oncogenic HPV types (HPV-6 and -11) in addition to the oncogenic HPV-16 and -18 types. Both vaccines have high efficacy (around 98%) against vaccine type-related HPV infections.^8,9^ Protection against oncogenic HPV types other than the vaccine type appears to be higher for the AS04-HPV-16/18 than the 4vHPV, as reported in their respective clinical trials.^10-13^

Both vaccines were originally developed for a 3-dose (3D) administration. Different studies with the AS04-HPV-16/18 have shown that 2-dose (2D) vaccination of 9-to-14-year-old girls was immunologically non-inferior to 3D vaccination of 15-to-25-year-old women.^14-16^ Recent evaluations also indicate a long-term sustention of antibody titres in this 9-to-14-year-old girls group up to 5 y after vaccination.^17,18^ All together, these studies suggest that a 2D schedule is sufficient for vaccination of 9-to-14-year-old girls. The vaccination schedule thus depends on the age of the vaccine recipient and on the license approved by the country.

The World Health Organization (WHO) recommends HPV vaccination to be included in national immunisation programmes in countries where CC and HPV-related diseases prevention is a public health priority and where vaccine introduction is programmatically and financially feasible and provided that cost-effectiveness of vaccination strategies in the country or region is considered.^19^ Currently, no routine HPV vaccination is in place in the Philippines. The Philippine authorities were one of the first to have approved the 2D schedule for the AS04-HPV-16/18 for the vaccination of girls from age 9 to 14 y inclusive, in January 2014.^20^ The use of a 3D regimen remains however recommended for use in girls aged 15 y and above.^21^ Recently, the Philippine authorities also approved the use of a 2D schedule for the 4vHPV.^22^ A 2D vaccination regimen could ease the implementation of the vaccination program and hence potentially increase uptake and completion rates.^17^ The 2D schedule would also reduce costs compared with a 3D schedule, which, if implemented, could be beneficial in countries like the Philippines where healthcare budgets are limited.

Information about the value for money, the budget and public health impact of available HPV vaccines in the Philippines may support decisions and choices about the country's vaccination program.

The objective of this study is to evaluate the effect of vaccination on CC-related and genital-warts-related disease burden and the cost-effectiveness of the 2D-AS04-HPV-16/18 compared with the 2D-4vHPV for universal vaccination of 13-year-old girls, in addition to the current screening program, in the Philippines.

## Results

### Model validation

This model, adapted to the Philippine settings, adequately reproduced age-dependent CC incidence (when compared with the 1998–2002 observed registry data in Manila - Additional File 1A) and age-dependent CC mortality (compared with CC GLOBOCAN 2008/2012 data reported for the Philippines - Additional File 1B). The model also adequately reproduced the age-dependent incidence of genital warts as reported in Japan (Additional File 1C).

### Base case

Table 1 shows the results of the base-case analysis.

**Table 1.** Results of the base-case analysis of a single-cohort of girls aged 13 (n = 986,910) (2D-AS04-HPV-16/18 *versus* 2D-4vHPV).

	Screening only	2D-AS04-HPV-16/18	2D-4vHPV	Difference
Number of cases				
CIN1 screening-detected	4,889	2,747	3,194	−447
CIN2/3 screening-detected	1,083	283	483	−200
Genital warts	17,380	17,381	4,435	12,946
Cervical cancer cases	10,539	2,412	3,398	−986
Cervical cancer deaths	4,250	981	1,380	−399
Undiscounted costs (PHP)				
Screening	549,870,626	550,417,467	552,277,351	−1,859,884
Vaccine cost	0	1,973,820,000	1,973,820,000	0
CIN1 treatment	26,798,999	15,062,654	17,649,517	−2,586,863
CIN2/3 treatment	45,608,607	12,052,878	20,553,054	−8,500,177
Genital warts	234,056,248	234,061,229	59,715,609	174,345,619
Cervical cancer	14,543,440,805	3,314,736,829	4,671,809,861	−1,357,073,032
Total costs	15,399,775,286	6,100,151,057	7,295,825,393	−1,195,674,336
Discounted results				
Total costs (PHP)	4,011,380,999	3,191,919,185	3,420,019,020	−228,099,835
Life-years	21,765,038	21,767,728	21,767,413	315
QALYs	21,759,744	21,766,305	21,765,749	555

CIN, cervical intraepithelial neoplasia; 4vHPV, HPV-6/11/16/18 L1 virus-like particle vaccine; AS04-HPV-16/18, HPV-16/18 AS04-adjuvanted vaccine; PHP, Philippine peso; QALY, quality-adjusted life year

The 2D-AS04-HPV-16/18 prevented more CC cases and deaths, screening-detected cervical intraepithelial neoplasia grade 1 (CIN1) and grade 2/3 (CIN2/3) than the 2D-4vHPV at a 100% vaccination coverage rate. The 2D-4vHPV prevented more cases of genital warts than the 2D-AS04-HPV-16/18.

The predicted number of life-years and quality-adjusted life years (QALYs) gained, after discounting, were higher with the 2D-AS04-HPV-16/18 than the 2D-4vHPV with a difference of 315 and 555, respectively.

The estimated savings in treatment costs as a result of the reduction in CIN (all grades) and CC cases with the 2D-AS04-HPV-16/18 would be expected to exceed the estimated savings in the treatment costs from the reduction in genital warts cases with the 2D-4vHPV. Thus, overall discounted treatment costs would be expected to be 228.1 million Philippine pesos (PHP) lower with the 2D-AS04-HPV-16/18 than the 2D-4vHPV for a vaccination coverage rate of 100%.

Consequently, the 2D-AS04-HPV-16/18 could be considered dominant, as it would be expected to both improve public health and reduce costs compared with the 2D-4vHPV.

### Sensitivity analyses

The parameters impacting the most on the QALY difference were, in order of importance: the proportion of non-vaccine HPV types in CC, the incidence of HPV oncogenic infection, the vaccine efficacy against non-vaccine HPV types, the HPV-6/11 types distribution in genital warts and the disutility associated with HPV disease (see Additional File 2A). Regarding the cost difference, the order of most-impacting parameters were: the HPV-16/18 types distribution in CC, the cost of vaccine, the screening-associated costs, the vaccine efficacy against non-vaccine HPV types and the cost of treatment of genital warts (see Additional File 2B). The only impact of the change in discount rate from 3% to 1.5% on QALY impact was limited to a switch between the ‘distribution of HPV-16/18 in CC’ and ‘utility loss with HPV disease’ parameters (see Additional File 2C). Regarding the impact on costs, the vaccine efficacy against non-vaccine HPV types and the incidence of oncogenic HPV infection in the general population became more influential. The order of influence of the CC treatment costs and the genital-warts costs are also swapped when changing the discount rate (See Additional File 2D).

### Probabilistic sensitivity analyses

**Figure 1.**
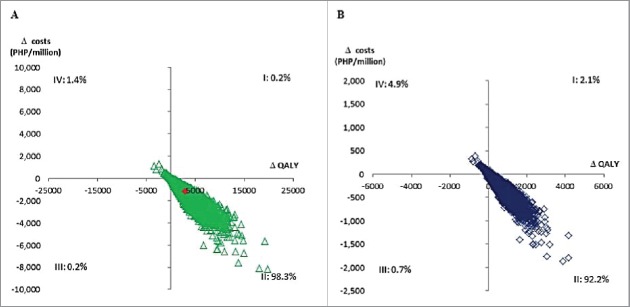
Probabilistic sensitivity analyses comparing AS04-HPV-16/18 with 4vHPV (A) without and (B) with discounting. PHP, Philippine peso; QALY, quality-adjusted life year.

The results of the probabilistic sensitivity analysis were plotted on a cost-effectiveness plane (Fig. 1). Without discounting, 98.3% of the replicates were in the second/dominant quadrant (cost-savings and QALY increase, AS04-HPV-16/18 vs. 4vHPV), while 1.4% of the replicates were in the fourth quadrant (cost-increase and QALY loss, AS04-HPV-16/18 vs. 4vHPV). With 3.5% discounting, 92.2% of the replicates were in the second/dominant quadrant and 4.9% of the replicates were in the fourth quadrant.

## Discussion

This study is the first to compare the cost-effectiveness of 2D-AS04-HPV-16/18 vaccine with 2D-4vHPV vaccine, in addition to screening, in the Philippines. The results estimate that the 2D-AS04-HPV-16/18 would generate more QALYs (i.e., increase health of the population) and be less costly than the 2D-4vHPV, with a probability of 92.2%. This reflects the effect of the higher protection against non-vaccine oncogenic HPV types reported for the AS04-HPV-16/18, which would provide greater protection against CIN and CC compared with the 4vHPV.^8,9,12,23,24^ The estimated reduction in treatment costs for CIN and CC with the 2D-AS04-HPV-16/18 would be expected to outweigh the effect of the reduction in genital-warts-associated costs with the 2D-4vHPV. The results presented here are consistent with a previous study of HPV vaccination in Taiwan using the same Markov model applied in a 3D setting, which also estimated that the AS04-HPV-16/18 would be dominant over the 4vHPV.^25^

Our analysis has limitations. As the model is static, it cannot take into account effects on disease transmission and thus cannot quantify any level of herd protection. Administration costs and the cost of logistics and storage for HPV vaccination were not included in the model, due to a lack of currently available data on these costs. This may have under-estimated the total programmatic costs of HPV vaccination. The model also excluded indirect costs, such as lost productivity due to time away from work. It is likely that the estimated lower number of CC cases remaining for the AS04-HPV-16/18 would also result in lower indirect costs. The model assumes a 100% vaccination coverage rate, which may be unrealistic in a real-world setting. However, this vaccination coverage rate was chosen to reflect the maximum possible achievable benefit associated with vaccination. Coverage rates of a different order will result in a linear decline in number of cases and associated cost offset. The resulting incremental cost-effectiveness ratio (ICER), however, is not affected by a different vaccination coverage since the model is fully static.^26^

Due to the lack of data on the incidence of genital warts in Filipino women in the general population, we used the best available data from the same region, which in this case were Japanese data. Although the incidence of genital warts was reported to be low in Japan compared with Western countries such as the United Kingdom and the United States, the expert panel who evaluated the input data for this model were of the opinion that these data were the most appropriate to be used for this analysis. The influence of a much higher genital warts burden in the Philippines has not been explored in this analysis due to the lack of reliable data but may have had a relatively strong influence on the results as could be inferred from the one-way sensitivity analysis on the impact of HPV-6/11 distribution in genital warts.

The results of this analysis suggest that the addition of 2D-AS04-HPV-16/18 vaccination to the current screening program in the Philippines would generate more QALYs and be less costly than using the 2D-4vHPV.

## Methods

### Model

#### Model structure

A static Markov cohort model with annual cycles was used to estimate the costs and health benefits of adding HPV vaccination to screening over 95 cycles (lifetime of the cohort). The model has been previously published and was adapted for this study to the Philippine setting by applying country-specific data on epidemiology, screening practice and costs.^27^

The model structure, which replicates the natural history of HPV infection, is summarised in Additional File 3. A series of health states represent the natural history of HPV infection. Subjects move between health states at each cycle according to fixed annual transition probabilities. Subjects in the cohort entering the model are assumed to be HPV-naive for oncogenic HPV infection [NoHPV]. At each cycle, a subject may remain in the same health state or become infected with low-risk HPV [HPVlr] or oncogenic HPV [HPVonc]. Subjects infected with low-risk HPV may then develop genital warts [genital wart] or CIN1 [CIN1lr], which may be detected and treated or may spontaneously clear (back to [NoHPV]). Subjects infected with oncogenic HPV may develop CIN1 [CIN1onc], which in turn may evolve into CIN grade 2/3 [CIN23], then progress to persistent CIN2/3 [Persistent CIN23], and then to cervical cancer [Cancer]. Cases of cervical cancer may be treated and cured [Cancer cured], or result in the death of the subject from cancer [Death cancer].

Screening was modeled by including a proportion of lesions that are detected by screening, based on the screening coverage and testing sensitivity, and applying different transition probabilities to subjects with detected lesions (‘det’). Subjects with detected lesions at each disease stage are in a different “detected” health state, with higher costs incurred for follow-up and treatment and a lower modified probability of progressing to a more advanced state if treatment is successful than the equivalent undetected state (because they receive medical follow-up and treatment).

Vaccination was modeled by modifying the transition probability for becoming infected with oncogenic or non-oncogenic HPV. Vaccination coverage rate was assumed to be 100% to reflect the maximum achievable benefit of vaccination.

#### Input data

Philippine specific data were used wherever available. However, there were certain parameters for which no local data were available. In such instances, data from another Asian country were used and validated by a group of key clinical experts who have extensive experience in cervical cancer care and have reviewed them to ensure the data were suitable for use. The group of key clinical experts agreed on the data inputs and assumptions used in the model during a round-table discussion held on July 10, 2013 in Manila, as part of a 2-round Delphi panel with a primary focus on cost data collection.

##### Demographics

A cohort of 13-year-old girls for the year 2013 was considered in the model (n = 986,910). The cohort size was estimated using data from the National Statistical Coordination Board.^28^ Age-specific mortality rates for the general population of the Philippines were obtained from the WHO mortality database^29^ and are summarised in Table 2.

**Table 2.** Transition probabilities between model health states.

Health states	Transition probability	Source	Remarks
Age-specific mortality	0.00221–0.42078	WHO – Philippines life tables^29^	Published; Philippines-specific
***Oncogenic HPV infection***			
HPVonc to No HPV	0.293–0.553	Age-specific natural yearly clearance of HPVonc infection^32,44-46^	Published; disease-specific
HPVonc to CIN1	0.049	Yearly spontaneous progression from HPVonc to CIN1. Adjusted from Moscicki et al (2001)^44^	Published; disease-specific
HPVonc to CIN2/3	0	Assumption (at least 2 y needed to develop CIN2/3)	Delphi panel; Philippines-specific
CIN1onc to Cured	0.449	Natural yearly regression from CIN1onc to NoHPV^47,48^	Published; disease-specific
CIN1 to CIN2/3	0.16	Adjusted from 0.09 after calibration^45,47,48^	Published data and Expert opinion
CIN2/3 to Cured	0.227	Spontaneous regression from CIN2/3 to NoHPV within 1 y^45^	Published; disease-specific
CIN2/3 to CIN1onc	0	Spontaneous regression from CIN2/3 to CIN1 within 1 y Assumption	Delphi panel; Philippines-specific
CIN2/3 to persistent CIN2/3	0.114	Spontaneous progression from CIN2/3 to persistent CIN2/3 within 1 y ( = 1- CIN2/3_cured - CIN2/3_CIN1Onc - CIN2/3_cancer)	Delphi panel; Philippines-specific
Persistent CIN2/3 to cancer	0.008–0.88	Annual probability of transition, assumed 0.008 at year 20 with a yearly increase of 0.008	Delphi panel; Philippines-specific
% CIN2/3 detected undergoing treatment	1	Assumption	Delphi panel; Philippines-specific
CIN2/3 treatment success	0.90	Treatment success defined as subject returning to normal state i.e., no HPV after treatment^47^	Delphi panel; Philippines-specific
Cancer to Death from CC	0.146	Mortality of patients with CC (natural mortality + additional mortality). The 5-year CC survival rate of metro Manila residents is 45.4%^32,49^. The annual CC survival rate is calculated as 1–45.4%^(1/5) = 14.6%	Published; Philippines-specific
Cancer to Cured	0.114	% patients still alive after 5 y (assumed to be cured) and facing general population mortality. The 5-year CC mortality rate is 100%−45.4% = 54.6%. The annual CC mortality rate is calculated as 1-(1–0.454)^(1/5) = 11.4%	Delphi panel; Philippines-specific
***Low-risk HPV infection***			
HPVlr to No HPV	0.516	Assumption - Natural yearly regression from low-risk HPV infection and genital warts^50^	Delphi panel; Philippines-specific
HPVlr to GW	0.0001–0.9865874	Yearly spontaneous progression from HPVlr infection to genital warts as based on genital warts incidence data from Japan^39^	Published; disease-specific
HPVlr to CIN 1	0.036	Yearly spontaneous progression from low-risk HPV infection to CIN1^47^	Published; disease-specific
% GW resistant	0.350	Proportion of treated genital warts resistant to initial treatment^33^	Published; disease-specific
CIN1lr to No HPV	0.50	Yearly natural regression from low-risk CIN1 to no HPV^47^	Published; disease-specific

CC, cervical cancer; CIN, cervical intraepithelial neoplasia; GW, genital warts; HPV, human papillomavirus; onc: oncogenic; lr, low-risk

##### Transition probabilities

The transition probabilities for low-risk and oncogenic HPV infection were obtained from published sources or calibrated,^25^ and are listed in Table 2.

##### Screening parameters

Screening coverage was set at 7.7% of women aged 18–69 y.^30^ Screening sensitivity was based on a systematic literature review and set at 58% for CIN1 lesions and 61% for CIN2/3 lesions.^31^

##### Costs

No published cost data were available for the Philippines. Therefore, cost data were obtained from a 2-round Delphi panel. The expert panel consisted of 6 members from Manila and Quezon City in the Philippines and was held in Manila on May and July 2013.

All costs related to the screening, treatment of pre-cancerous lesions and genital warts were provided as point estimates for both private and public healthcare settings in an urban area, by the members of the expert group. An average of private and public estimated costs was used for the base-case analysis.

CC treatment costs were based on an unpublished costing study performed by a member of the expert group in a hospital based in Manila.

The obtained cost figures were provided by experts during the first meeting and thereafter discussed by all experts through e-mail exchange and finally endorsed by all experts during a second meeting. All costs represent direct medical costs and were calculated on an annual basis for the year 2012–2013. Cost data are shown in Table 3. Price-per-dose parity was assumed for both vaccines.

**Table 3.** Annual costs of treatment and minimum/maximum values used for sensitivity analyses and vaccine price (results from Philippine Delphi panel).

	Annual cost (PHP)		
Parameter	Average	Minimum (Public)	Maximum (Private)
Cost of regular screening for subjects with negative pap smear	550	100	1,000
Cost of regular screening for positive pap smear subject, plus colposcopy/biopsy	1,425	1,200	1,650
Treatment cost of CIN1	4,500	3,000	6,000
Treatment cost of CIN2/3	34,000	16,000	52,000
Average yearly treatment cost for genital warts and resistant genital warts in females	8,786	5,000	20,000
Composite average yearly treatment costs accounting for each stage of CC	244,763	205,132	251,120
Price vaccine per dose^*^	1,000		

* Assumption

CC, cervical cancer; CIN, cervical intraepithelial neoplasia; Pap, Papanicolaou; PHP, Philippine peso

##### Vaccine effectiveness

The model uses vaccine effectiveness against CC on incident oncogenic HPV infection and vaccine effectiveness against genital warts on incident low-risk HPV infection. These were thereafter adjusted for each lesion.

A proxy for overall vaccine effectiveness (VE), including protection against non-vaccine HPV types, was calculated by combining the vaccine efficacy against each HPV type (VE_i_) with the proportion of each HPV type within each type of lesion (%HPV_i_), according to the following equation:

$$VE\;=\sum_{i}^{\;}\%\;HPV_{i}*VE_{i}$$

Vaccine efficacy data were taken from clinical trials.^8,9,12,23,24^ HPV-type distribution data were taken from the Institut Català d'Oncologia (ICO) HPV Center database for CIN and CC.^30^ For genital warts, a weighted average was calculated from HPV distribution data retrieved from 3 epidemiological studies including more than 100 female patients each. See Table 4 for details.

**Table 4.** Vaccine effectiveness against each type of lesion and disutilities.

Parameter	HPV type distribution (%)	AS04-HPV-16/18 vaccine efficacy (95% CI)	4vHPV vaccine efficacy (95% CI)
CIN1			
HPV-16/18	25.7% (ICO HPV center - Asia continent)^30^	98%^8^	98%^9^
Cross protection^*^	50.1% (ICO HPV center - Asia continent)^30^	48% (29–62)^8,23^	23% (8–36)^12^
HPV-6/11	3.1% (ICO HPV center - Asia continent)^30^	0%	98%^9^
*Overall effectiveness*		***49.1%***	***39.7%***
Genital warts			
HPV-6/11	76.7% (weighted average from^51-53^)	0%	98%^9^
*Overall effectiveness*		***0.0%***	***75.2%***
CIN2/3			
HPV-16/18	42.4% (ICO HPV center - Asia continent)^30^	98%^8^	98%^9^
Cross protection^*^	50.2% (ICO HPV center - Asia continent)^30^	68% (46–82)^8,24^	33% (6–52)^12^
*Overall effectiveness*		***75.9%***	***58.1%***
Cervical cancer			
HPV-16/18	63.7 (ICO HPV center - Philippines)^30^	98%^8^	98%^9^
Cross protection^*^	24.8 (ICO HPV center - Philippines)^30^	68% (46–82)^8,24^	33% (6–52)^12^
*Overall effectiveness*		***79.4%***	***70.6%***

CI, confidence interval; CIN, cervical intraepithelial neoplasia; HPV, human papillomavirus.

* Cross-protection against HPV types 31/33/35/39/45/51/52/56/58/59

For both vaccines, the analysis used data on vaccine efficacy in girls and women who were DNA-negative and seronegative for the relevant HPV type at study entry (i.e., HPV-naive). These data are the most representative of vaccine efficacy among girls before the onset of sexual activity and are therefore the most relevant to the vaccination of 13-year-old girls in the Philippines.

The vaccine efficacy was assumed to be the same for both the 2D vaccines as non-inferiority in the immune response has been observed with the 2D and 3D schedule for the AS04-HPV-16/18v.^14-18^ Details of vaccine effectiveness data inputs are shown in Table 4.

##### Disutilities

Since country-specific utility data for the Philippines were lacking, published disutility data from other evaluations of HPV vaccination were applied in this analysis.^32-37^ See Additional File 5 for details.

#### Model validation

The model was validated against observed local/regional epidemiological outcomes. The CC incidence was retrieved from the 1998–2002 cancer registry data in Manila as reported by the Philippine Cancer Society.^38^ CC mortality was retrieved from GLOBOCAN 2008/2012 for the Philippines.^1^ As the incidence of genital warts was not available for the Philippines, Japanese data, closest Asian country where data were available, was validated as the most appropriate source of data by the Delphi panel.^39^

### Cost-effectiveness analysis

#### Discount rate

In the base case, all costs and outcomes were estimated non-discounted and discounted at 3.5% per year, as recommended by the Philippine Ministry of Health.^40^

#### Perspective

The analysis was conducted from the perspective of the Philippine government. Only direct medical costs (hospitalisation, screening tests and procedures, vaccine costs) were included.

#### Interventions

The base-case analysis compared vaccination of 13-year-old girls with either the 2D-AS04-HPV-16/18 or the 2D-4vHPV, in addition to screening. Vaccination coverage was assumed to be 100% with all subjects completing the full vaccination course.

#### Outcomes

The model estimated the lifetime number of cases of CC, CIN1, CIN2/3 and genital warts, associated costs and QALYs for a 13-year-old girls cohort vaccinated with either the 2D-AS04-HPV-16/18 or 2D-4vHPV. Incremental cost, QALY and cost-effectiveness were derived from these estimates.

Cost-effectiveness thresholds from 1xGDP (gross domestic product)/capita ( = “highly cost-effective”) to 3xGDP/capita ( = “cost effective”) were used, as recommended by the World Health Organization (WHO).^41^ GDP/capita in the Philippines was PHP 118,295 in 2013.^42^

### Sensitivity analyses

Both one-way and probabilistic sensitivity analyses were conducted.

#### One-way sensitivity analyses

One-way sensitivity analyses evaluated the effect on the results of variability in the model parameters. The key parameters were varied by ±20% from the base-case values used except for vaccine efficacy and cost parameters. For vaccine efficacy, the reported 95% confidence intervals were used (see Table 4). For the treatment cost, the government hospital cost was used as the lower limit and the cost at a private institution as the upper limit (see Table 3).

A specific sensitivity analysis explored the effect of using a discount rate of 1.5% for costs and outcomes as recently recommended by the National Institute for Clinical Excellence (NICE) in the United Kingdom for healthcare interventions with long-lasting health benefits.^43^ Changing the discount rate to a lower value gives more weight to benefits further away such as reductions in CC cases which may better reflect the time preference of subjects or decision-makers, rather than a high discount rate.

#### Probabilistic sensitivity analysis

A probabilistic sensitivity analysis was conducted using Monte Carlo simulation with the software package @*Risk* (Palisade Corporation, Ithaca, NY). Distributions were assigned to input parameters by using a normal distribution when confidence intervals were reported and uniform distribution when no range was available (see Additional File 4 for details). A total of 10,000 iterations were sampled from the assigned distribution.

## Supplementary Material

Supplementary files

## Notes

*Cervarix*™ is a trademark of the GSK group of companies.

## Abbreviations

2D: 2-dose
3D: 3-dose
4vHPV: HPV-6/11/16/18 L1 virus-like particle vaccine
AS04-HPV-16/18: HPV-16/18 AS04-adjuvanted vaccine
CC: cervical cancer
CI: confidence interval
CIN: cervical intraepithelial neoplasia
CIN1/2/3: cervical intraepithelial neoplasia grade 1/2/3
Det: detected lesions
GDP: gross domestic product
HPV: human papillomavirus
ICO: Institut Català d'Oncologia
lr: low-risk
NICE: National Institute for Clinical Excellence
onc: oncogenic
Pap: Papanicolaou
PHP: Philippine peso
QALY: quality-adjusted life year
VE: vaccine efficacy
WHO: World Health Organization
