# Supplementary material for: Cost-effectiveness analysis of AS04-adjuvanted human papillomavirus 16/18 vaccine compared with human papillomavirus 6/11/16/18 vaccine in the Philippines, with the new 2-dose schedule
Source: Hum Vaccin Immunother. 2017 Jan 11;13(5):1158–66. doi: 10.1080/21645515.2016.1269991 (PMC5443386; doi:10.1080/21645515.2016.1269991)
Supplement: Supplementary files [file khvi-13-05-1269991-s001.zip › 2016HV0328R1-s01.pdf]

Additional File 1 – Modelled CC incidence (A), CC mortality (B) and GW incidence (C) against observed epidemiological data [1-3]

(A)

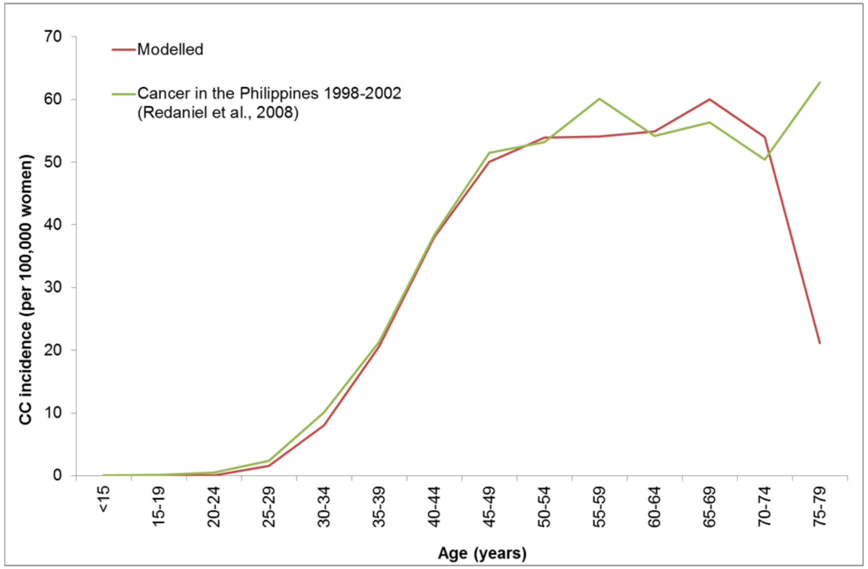

(B)

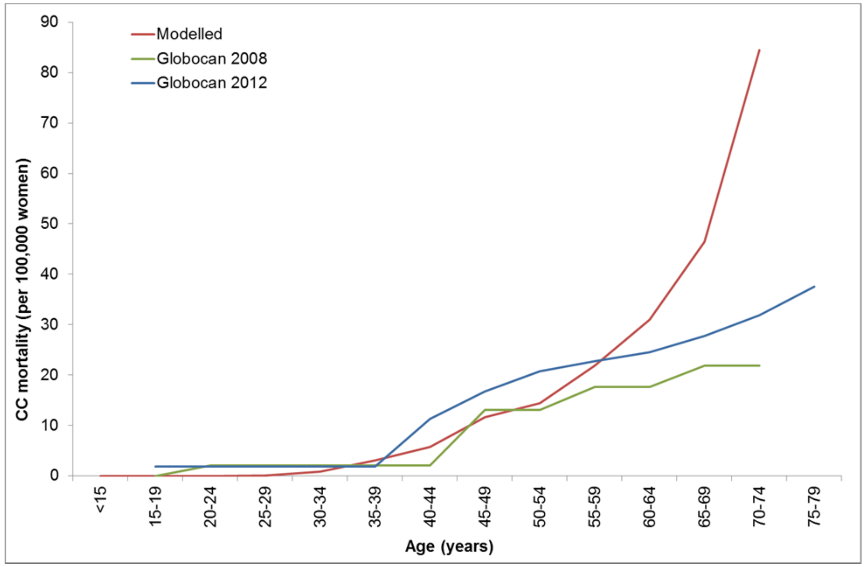

(C)

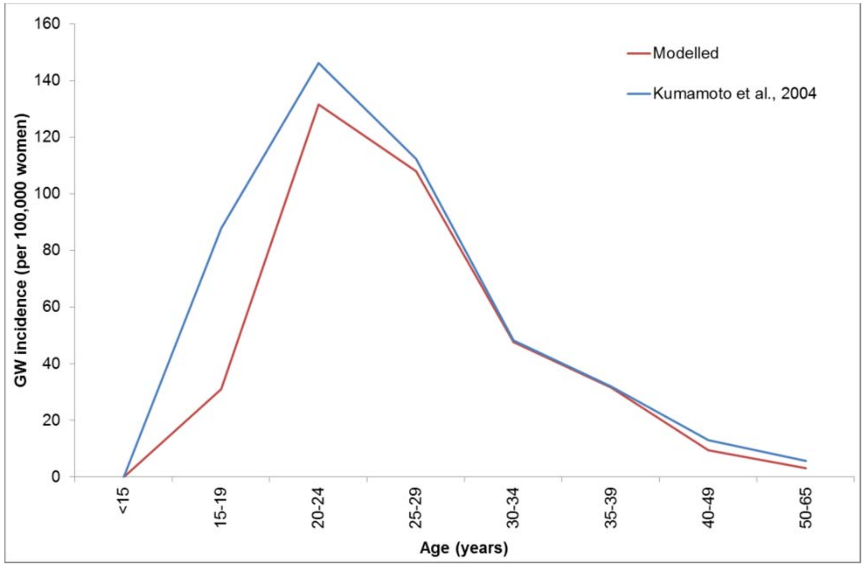

References:

1. Redaniel, Maria Theresa M., Laudico, Adriano V., Lumague, Maria Rica Mirasol., Mapua, Cynthia A., Patama, T., and Pukkula, Eero. Cancer in the Philippines; Vol. IV Part 1 - Cancer Incidence 1998-2002. Manila: Philippine Cancer Society; 2008.

2. Ferlay J, Soerjomataram I, Ervik M, Dikshit R, Eser S, Mathers C, Rebelo M, Parkin DM, Forman D, and Bray F. GLOBOCAN 2012 v1.0, Cancer incidence and mortality worldwide [Internet]. Lyon, France: International Agency for Research on Cancer; 2013 [cited Jan 31, 2014]. Available from: http://globocan.iarc.fr.

3. Kumamoto Y, Tsukamoto J, Sugiyama T, Akaza H, Noguchi M, Naya A, Kamidono S, Usui T, Kagawa S, Tanaka M, et al. National surveillance of sexually transmitted diseases of Japan in 2002 (Article in Japanese). Japanese Journal of Sexually Transmitted Diseases 2004; 15: 17-45.
