# Supplementary material for: Cost-effectiveness analysis of AS04-adjuvanted human papillomavirus 16/18 vaccine compared with human papillomavirus 6/11/16/18 vaccine in the Philippines, with the new 2-dose schedule
Source: Hum Vaccin Immunother. 2017 Jan 11;13(5):1158–66. doi: 10.1080/21645515.2016.1269991 (PMC5443386; doi:10.1080/21645515.2016.1269991)
Supplement: Supplementary files [file khvi-13-05-1269991-s001.zip › 2016HV0328R1-s02.pdf]

Additional file 2 - One-way sensitivity analysis – Tornado graphs

One-way sensitivity analysis on QALY and cost differences at discount rate 3.5% (A and B) and at discount rate 1.5% (C and D)

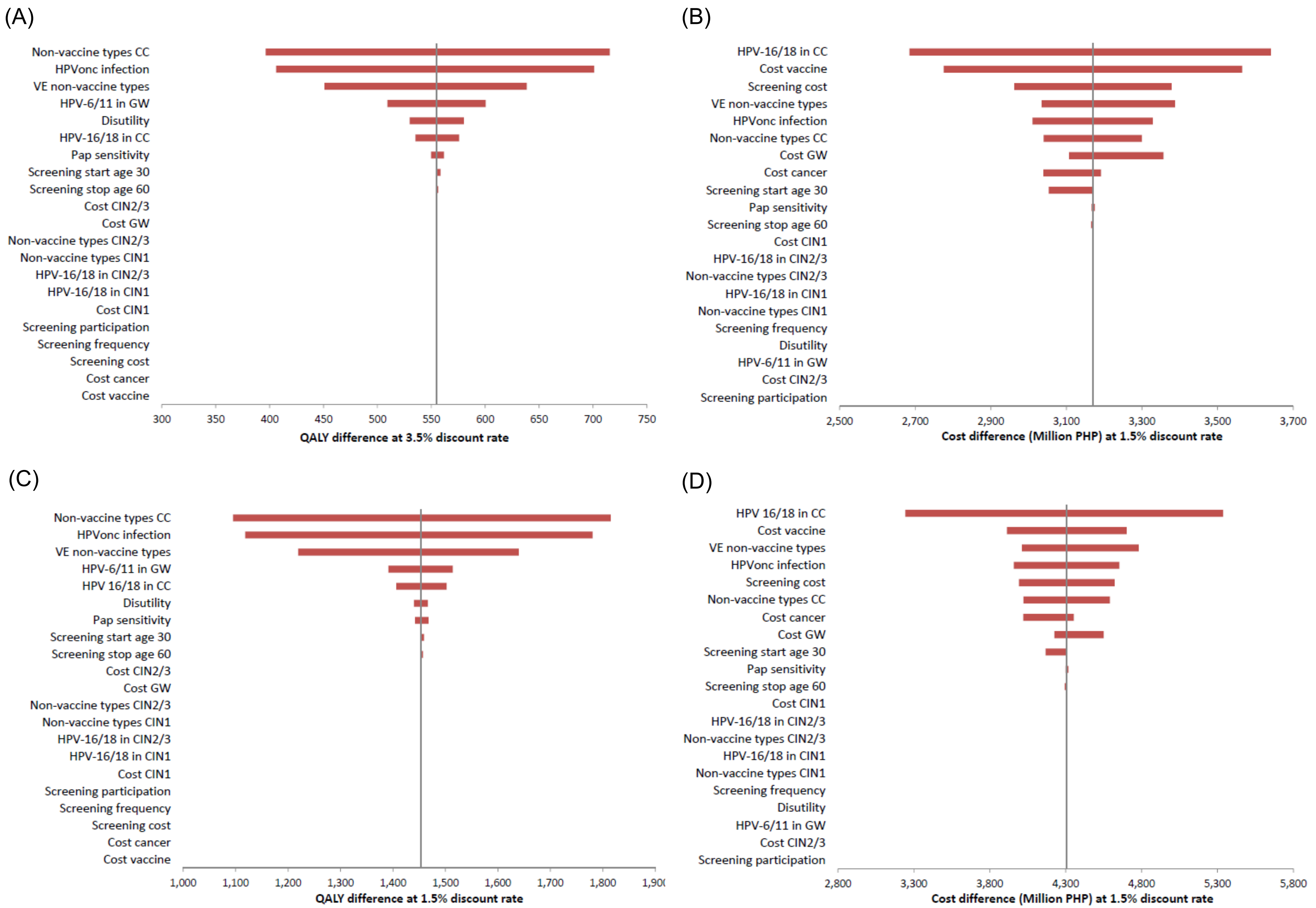

CC, cervical cancer; CIN1/2/3, cervical intraepithelial neoplasia grade 1/2/3; GW, genital warts; HPV, human papillomavirus; onc, oncogenic; PHP, Philippine peso; QALY, quality-adjusted life-year; VE, vaccine effectiveness
