# Supplementary material for: Cost-effectiveness analysis of AS04-adjuvanted human papillomavirus 16/18 vaccine compared with human papillomavirus 6/11/16/18 vaccine in the Philippines, with the new 2-dose schedule
Source: Hum Vaccin Immunother. 2017 Jan 11;13(5):1158–66. doi: 10.1080/21645515.2016.1269991 (PMC5443386; doi:10.1080/21645515.2016.1269991)
Supplement: Supplementary files [file khvi-13-05-1269991-s001.zip › 2016HV0328R1-s03.pdf]

Additional File 3 – Model structure

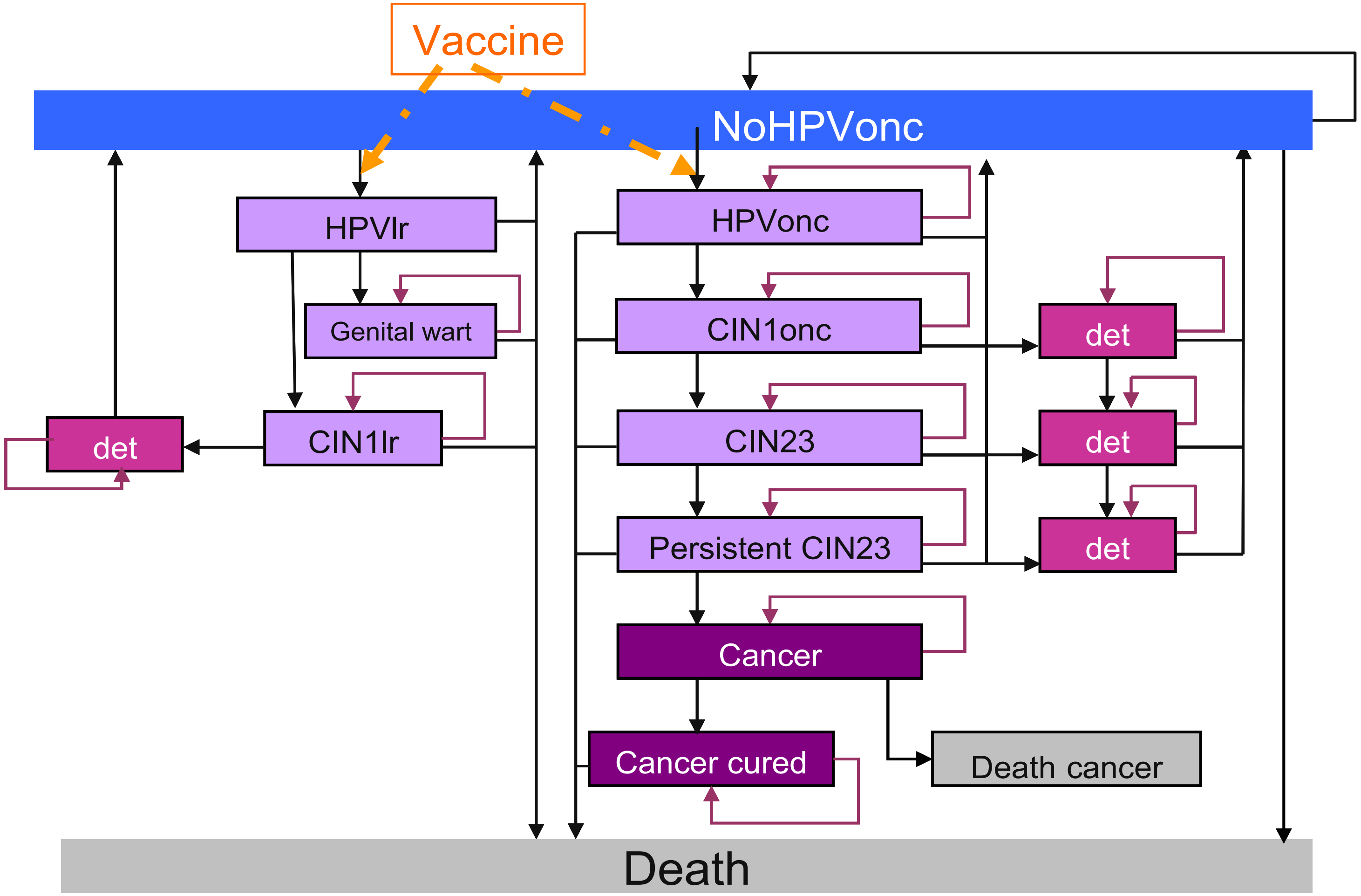

Reproducing with permission: Figure 1 - Modelling the economic value of cross- and sustained-protection in vaccines against cervical cancer. Demarteau N, Standaert B. Journal of Medical Economics; 13(2) 2010

CIN, cervical intraepithelial neoplasia; det, detected (subjects with disease detected through screening: same pathways but different probabilities); HPV, human papillomavirus; lr, low-risk; onc, oncogenic
