# Supplementary material for: Cost-effectiveness analysis of AS04-adjuvanted human papillomavirus 16/18 vaccine compared with human papillomavirus 6/11/16/18 vaccine in the Philippines, with the new 2-dose schedule
Source: Hum Vaccin Immunother. 2017 Jan 11;13(5):1158–66. doi: 10.1080/21645515.2016.1269991 (PMC5443386; doi:10.1080/21645515.2016.1269991)
Supplement: Supplementary files [file khvi-13-05-1269991-s001.zip › 2016HV0328R1-s04.docx]

# Additional file 4 - Values and distributions used for probabilistic sensitivity analysis

| **Health states** | **Distribution** | **Source** |
| --- | --- | --- |
| Age-specific mortality | Uniform distribution (0.002–0.337; 0.003–0.505) | Assumption. Multiplied at each age by a uniform distribution from 0.8– 1.2 |
| ***Oncogenic HPV infection*** | | |
| HPVonc to No HPV | Uniform distribution (0.234–0.442; 0.352–0.664) | Assumption. Multiplied at each age by a uniform distribution from 0.8– 1.2 |
| HPVonc to CIN1 | Normal distribution 0.049 (SD 0.009) | Moscicki et al, 2001^1^ |
| HPVonc to CIN2/3 | Fix (0) | Assumption |
| CIN1onc to Cured | Normal distribution 0.449 (SD 0.142) | Sanders et al, 2003^2^; Van de Velde et al, 2007^3^ |
| CIN1 to CIN2/3 | Normal distribution 0.160 (SD 0.021) | Melnikow et al, 1998^4^; Sanders et al, 2003 Sanders, 2003 1776 /id}; Van de Velde et al, 2007^3^ |
| CIN2/3 to Cured | Normal distribution 0.227 (SD 0.058) | Melnikow et al, 1998^4^ |
| CIN2/3 to CIN1onc | Fix (0) | Assumption |
| CIN2/3 to persistent CIN2/3 | Uniform distribution 0.091-0.137 | Melnikow et al, 1998^4^ |
| Persistent CIN2/3 to cancer | Uniform distribution 0.006– 0.704; 0.010– 1.056 | Assumption. Multiplied at each age by a uniform distribution from 0.8– 1.2 |
| % CIN2/3 detected undergoing treatment | Fix (1) | Assumption |
| CIN2/3 treatment success | Uniform distribution (0.72– 1) | Assumption based on Delphi panel |
| Cancer to Death from CC | Uniform distribution (0.117–0.175) | Assumption |
| Cancer to Cured | Uniform distribution (0.091–0.137) | Assumption |
| ***Low risk HPV infection*** | | |
| HPVlr to No HPV | Uniform distribution (0.413–0.619) | Richardson et al, 2003^5^ |
| HPVlr to GW | Uniform distribution (0.00008–1) | Multiplied at each age by a uniform distribution from 0.8-1.2; Kumamoto et al, 2004 ^6^ |
| HPVlr to CIN 1 | Normal distribution 0.036 (SD 0.005) | Van de Velde et al., 2007^3^ |
| % GW resistant | Uniform distribution (0.280–0.420) | Woodhall et al., 2011^7^ |
| CIN1lr to No HPV | Normal distribution (0.50 SD 0.145) | Van de Velde et al., 2007^3^ |
| Cost of regular screening for subjects with negative pap smear | Uniform distribution (440–660) | Delphi panel |
| Cost of regular screening for positive pap  smear subject, plus colposcopy/biopsy | Uniform distribution (1 140–1 710) | Delphi panel |
| Treatment cost of CIN1 | Uniform distribution (3 600–5 400) | Delphi panel |
| Treatment cost of CIN2/3 | Uniform distribution (27 200–40 800) | Delphi panel |
| Average yearly treatment cost for GW and resistant GW in females | Uniform distribution (7 029–10 543) | Delphi panel |
| Composite average yearly treatment costs accounting for each stage of CC | Uniform distribution (195 810–293 716) | Delphi panel |
| Price vaccine per dose (both vaccine) | Fix (1 000) | Assumption |
| ***Disutilities*** | | |
| No HPV | Fix (0) |  |
| HPV, CIN1, CIN2/3 undetected | Fix (0) |  |
| CIN1 detected | Uniform distribution (0.010–0.015) | Insinga et al., 2005; ^8^ Myers et al., 2004^9^ |
| CIN2/3 detected | Uniform distribution (0.010–0.015) | Insinga et al., 2005; ^8^ Myers et al., 2004^9^ |
| GW | Uniform distribution (0.014–0.022) | Gold et al., 1998 ^10^; Myers et al., 2004^9^ |
| Cancer | Uniform distribution (0.218–0.328) | Insinga et al., 2005; ^8^ Myers et al., 2004^9^ |
| Cancer cured | Uniform distribution (0.050–0.074) | Insinga et al., 2005; ^8^ Myers et al., 2004^9^ |
| Death | Fix (1) |  |
| ***Screening effectiveness*** | | |
| CIN1 detected | Normal distribution 0.58 (SD 0.045) | Fahey et al., 1995^11^ |
| CIN2/3 detected | Normal distribution 0.61 (SD 0.045) | Fahey et al., 1995^11^ |
| Vaccine effectiveness | | |
| AS04-HPV-16/18 vaccine effectiveness against 16/18 | Fix (0.980) | Paavonen et al., 2009^12^ |
| AS04-HPV-16/18 vaccine effectiveness against other 10 HPV-types in CIN1 | Normal distribution 0.477 (SD 0.083) | Paavonen et al., 2009^12^; Tjalma et al., 2009^13^ |
| AS04-HPV-16/18 vaccine effectiveness against other 10 HPV-types in CIN2/3 | Normal distribution 0.684 (SD 0.083) | Paavonen et al., 2009^12^;Skinner et al., 2009^14^ |
| AS04-HPV-16/18 vaccine effectiveness against other 10 HPV-types in CC | Normal distribution 0.684 (SD 0.083) | Paavonen et al., 2009^12^;Skinner et al., 2009^14^ |
| 4vHPV vaccine effectiveness against 16/18 | Fix (0.980) | The FUTURE II Study Group, 2007^15^ |
| 4vHPV vaccine effectiveness against other 10 HPV-types in CIN1 | Normal distribution 0.231 (SD 0.072) | Brown et al., 2009^16^ |
| 4vHPV vaccine effectiveness against other 10 HPV-types in CIN2/3 | Normal distribution 0.332 (SD 0.111) | Brown et al., 2009^16^ |
| 4vHPV vaccine effectiveness against other 10 HPV-types in CC | Normal distribution 0.332 (SD 0.111) | Brown et al., 2009^16^ |
| 4vHPV vaccine effectiveness against HPV-6/11 | Normal distribution 0.980 (SD 0.065) | The FUTURE II Study Group, 2007^15^; Garland et al., 2007^17^; Villa et al., 2006^18^ |
| ***HPV type distribution*** | | |
| HPV-6/11 in CIN1 | Uniform distribution (0.025–0.037) | Institut Català d'Oncologia (ICO), 2014^19^ |
| HPV-16/18 in CIN1 | Uniform distribution (0.206–0.308) | Institut Català d'Oncologia (ICO), 2014^19^ |
| HPV-16/18 in CIN2/3 | Uniform distribution (0.339–0.509) | Institut Català d'Oncologia (ICO), 2014^19^ |
| HPV-16/18 in CC | Uniform distribution (0.510–0.637) | Aubin et al., 2008^20^; Garland et al., 2009^21^ |
| HPV-6/11 in GW | Normal distribution 0.762 (SD 0.125) | Institut Català d'Oncologia (ICO), 2014^19^ |

HPV, human papillomavirus; CC, cervical cancer; CIN, cervical intraepithelial neoplasia; GW, genital warts; lr, low risk; Pap, Papanicolaou; SD, standard deviation

## References

1. Moscicki AB, Hills N, Shiboski S, Powell K, Jay N, Hanson E, Miller S, Clayton L, Farhat S, Broering J, et al. Risks for incident human papillomavirus infection and low-grade squamous intraepithelial lesion development in young females. JAMA 2001 Jun 20; 285: 2995-3002. doi:10.1001/jama.285.23.2995.

2. Sanders GD, Taira AV. Cost-effectiveness of a potential vaccine for human papillomavirus. Emerg Infect Dis 2003 Jan; 9: 37-48. doi:10.3201/eid0901.020168.

3. Van de Velde N, Brisson M, Boily MC. Modeling human papillomavirus vaccine effectiveness: quantifying the impact of parameter uncertainty. Am J Epidemiol 2007 Apr 1; 165: 762-75. doi: 10.1093/jnci/djg037.

4. Melnikow J, Nuovo J, Willan AR, Chan BK, Howell LP. Natural history of cervical squamous intraepithelial lesions: a meta-analysis. Obstet Gynecol 1998 Oct; 92: 727-35.

5. Richardson H, Kelsall G, Tellier P, Voyer H, Abrahamowicz M, Ferenczy A, Coutlee F, Franco EL. The natural history of type-specific human papillomavirus infections in female university students. Cancer Epidemiol Biomarkers Prev 2003 Jun 1; 12: 485-90.

6. Kumamoto Y, Tsukamoto J, Sugiyama T, Akaza H, Noguchi M, Naya A, Kamidono S, Usui T, Kagawa S, Tanaka M, et al. [National surveillance of sexually transmitted diseases of Japan in 2002]. Japanese Journal of Sexually Transmitted Diseases 2004; 15: 17-45.

7. Woodhall SC, Jit M, Soldan K, Kinghorn G, Gilson R, Nathan M, Ross JD, Lacey CJ. The impact of genital warts: loss of quality of life and cost of treatment in eight sexual health clinics in the UK. Sex Transm Infect 2011 Oct; 87: 458-63. doi:10.1136/sextrans-2011-050073.

8. Insinga R, Glass A, Rush B. Health state transitions following an abnormal pap smear: implications for health utility assessment in cost-effectiveness analyses [Abstract W-02]. 22nd International Papillomavirus Conference & Clinical Workshop; 2005 Apr 30; Vancouver, BC, Canada. 2005.

9. Myers ER, Green S, Lipkus I. Patient preferences for health states related to HPV infection: visual analog scale versus time trade-off elicitation. [Abstract n° 542]. Twenty-First International Papillomavirus Conference; 2004 Feb 20; México City, México. 2004.

10. Gold MR, Franks P, McCoy KI, Fryback DG. Toward consistency in cost-utility analyses: using national measures to create condition-specific values. Med Care 1998 Jun; 36: 778-92.

11. Fahey MT, Irwig L, Macaskill P. Meta-analysis of Pap test accuracy. Am J Epidemiol 1995 Apr 1; 141: 680-9.

12. Paavonen J, Naud P, Salmeron J, Wheeler C, Chow SN, Apter D, Kitchener H, Castellsague X, Teixeira J, Skinner S, et al. Efficacy of human papillomavirus (HPV)-16/18 AS04-adjuvanted vaccine against cervical infection and precancer caused by oncogenic HPV types (PATRICIA): final analysis of a double-blind, randomised study in young women. Lancet 2009 Jul 6; 374: 301-14. doi:10.1016/S0140-6736(09)61248-4.

13. Tjalma W, Paavonen J, Naud P, Wheeler CM, Chow SN, Apter D, Kitchener H, Castellsagué X, Teixeira JC, Skinner SR, et al. Efficacy of the HPV-16/18 AS04-adjuvanted vaccine against abnormal cytology and low-grade histopathological lesions in an oncogenic HPV-naïve population. [Abstract n° A-171-0004-01446 presented at the 16th International Meeting of the European Society for Gynaecological Oncology (ESGO), 11 - 14 Oct, Belgrade, Serbia]. Int J Gynecol Cancer 2009 Oct; 19: 1008.

14. Skinner R, Apter D, Chow SN, Wheeler C, Dubin G. Cross-protective efficacy of Cervarix against oncogenic HPV types beyond HPV 16/18: Final analysis of cross-protection - PATRICIA study [Abstract]. 25th International Papillomavirus Conference; 2009 May 8; Malmö, Sweden. International Papillomavirus Society; 2009. p. 469 Available from: <http://www.hpv2009.org/Abstractbok_hela%20webb%20low%5B1%5D.pdf>.

15. The FUTURE II Study Group. Quadrivalent Vaccine against Human Papillomavirus to Prevent High-Grade Cervical Lesions. The New England Journal of Medicine 2007 May 10; 356: 1915-27. doi:10.1056/NEJMoa061741.

16. Brown DR, Kjaer SK, Sigurdsson K, Iversen OE, Hernandez-Avila M, Wheeler CM, Perez G, Koutsky LA, Tay EH, Garcia P, et al. The impact of quadrivalent human papillomavirus (HPV; types 6, 11, 16, and 18) L1 virus-like particle vaccine on infection and disease due to oncogenic nonvaccine HPV types in generally HPV-naive women aged 16-26 years. J Infect Dis 2009 Apr 1; 199: 926-35. doi: 10.1086/597307.

17. Garland SM, Hernandez-Avila M, Wheeler CM, Perez G, Harper DM, Leodolter S, Tang GWK, Ferris DG, Steben M, Bryan J, et al. Quadrivalent Vaccine against Human Papillomavirus to Prevent Anogenital Diseases. N Engl J Med 2007 May 10; 356: 1928-43.

18. Villa LL. Vaccines against papillomavirus infections and disease. Rev Chilena Infectol 2006 Jun; 23: 157-63.

19. Institut Català d'Oncologia. ICO (Institut Català d'Oncologia) Information Centre on HPV and Cancer (HPV Information Centre). [accessed 2014 Feb 10]. <http://www.hpvcentre.net/dataquery.php>.

20. Aubin F, Pretet JL, Jacquard AC, Saunier M, Carcopino X, Jaroud F, Pradat P, Soubeyrand B, Leocmach Y, Mougin C, et al. Human papillomavirus genotype distribution in external acuminata condylomata: a Large French National Study (EDiTH IV). Clin Infect Dis 2008 Sep 1; 47: 610-5. doi: 10.1086/590560.

21. Garland SM, Steben M, Sings HL, James M, Lu S, Railkar R, Barr E, Haupt RM, Joura EA. Natural History of Genital Warts: Analysis of the Placebo Arm of 2 Randomized Phase III Trials of a Quadrivalent Human Papillomavirus (Types 6, 11, 16, and 18) Vaccine. J Infect Dis 2009 Mar 15; 199: 805-14. doi: 10.1086/597071.
