# Supplementary material for: Cost-effectiveness analysis of AS04-adjuvanted human papillomavirus 16/18 vaccine compared with human papillomavirus 6/11/16/18 vaccine in the Philippines, with the new 2-dose schedule
Source: Hum Vaccin Immunother. 2017 Jan 11;13(5):1158–66. doi: 10.1080/21645515.2016.1269991 (PMC5443386; doi:10.1080/21645515.2016.1269991)
Supplement: Supplementary files [file khvi-13-05-1269991-s001.zip › 2016HV0328R1-s05.docx]

# Additional File 5 - Disutility scores^1-6^

| **Health state** | **Value** |
| --- | --- |
| CIN1 | 0.0128 |
| CIN2/3 | 0.0128 |
| Cervical cancer | 0.273 |
| Cervical cancer cured | 0.062 |
| Genital warts | 0.018 |
| Normal utility | 1 |

CIN1/2/3, cervical intraepithelial neoplasia grade 1/2/3

## References

1. Goldie SJ, Kohli M, Grima D, Weinstein MC, Wright TC, Bosch FX, Franco E. Projected clinical benefits and cost-effectiveness of a human papillomavirus 16/18 vaccine. J Natl Cancer Inst 2004 Apr 21; 96: 604-15. doi: 10.1093/jnci/djh104.

2. Woodhall SC, Jit M, Soldan K, Kinghorn G, Gilson R, Nathan M, Ross JD, Lacey CJ. The impact of genital warts: loss of quality of life and cost of treatment in eight sexual health clinics in the UK. Sex Transm Infect 2011 Oct; 87: 458-63. doi:10.1136/sextrans-2011-050073.

3. Gold MR, Franks P, McCoy KI, Fryback DG. Toward consistency in cost-utility analyses: using national measures to create condition-specific values. Med Care 1998 Jun; 36: 778-92.

4. Stratton K, Durch J, Lawrence S. Vaccines for the 21st century: a tool for decisionmaking; Appendix 11: Human Papillomavirus.Washington, DC: National Academy Press; 2000.

5. Insinga R, Glass A, Rush B. Health state transitions following an abnormal pap smear: implications for health utility assessment in cost-effectiveness analyses [Abstract W-02]. 22nd International Papillomavirus Conference & Clinical Workshop; 2005 Apr 30; Vancouver, BC, Canada. 2005.

6. Myers ER, Green S, Lipkus I. Patient preferences for health states related to HPV infection: visual analog scale versus time trade-off elicitation. [Abstract n° 542]. Twenty-First International Papillomavirus Conference; 2004 Feb 20; México City, México. 2004.
